# An Analytical Model of Motion Artifacts in a Measured Arterial Pulse Signal—Part I: Accelerometers and PPG Sensors

**DOI:** 10.3390/s25185710

**Published:** 2025-09-12

**Authors:** Md Mahfuzur Rahman, Subodh Toraskar, Mamun Hasan, Zhili Hao

**Affiliations:** Department of Mechanical and Aerospace Engineering, Old Dominion University, Norfolk, VA 23529, USA; mrahm009@odu.edu (M.M.R.); stora002@odu.edu (S.T.); mhasa004@odu.edu (M.H.)

**Keywords:** arterial pulse measurement, accelerometers, baseline drift, dynamic systems, motion artifacts, transmission path, time-varying system parameters, PPG sensors

## Abstract

This paper, the first of two parts, presents an analytical model of motion artifacts (MAs) in measured pulse signals by accelerometers and photoplethysmography (PPG) sensors. As the transmission path from the true pulse signal in an artery to the sensor output (measured pulse signal), the tissue–contact–sensor (TCS) stack is modeled as a 1DOF (degree-of-freedom) system. MAs cause baseline drift of the mass and simultaneously time-varying system parameters (TVSPs) of the TCS stack. With arterial wall displacement and pulsatile pressure serving separately as the true pulse signal, an analytical model is developed to mathematically relate baseline drift and TVSP to a measured pulse signal. With assumed values of baseline drift and TVSPs, the numerical calculation is conducted in MATLAB. While baseline drift is low-frequency additive noise and can greatly swing a measured pulse signal, TVSP generates relatively small, abrupt distortion (e.g., 1% variation in heart rate and <5% change in pulse amplitude) but rides on each harmonic of the true pulse signal. By taking into account the full involvement of the transmission path in pulse measurement, this analytical model serves as a fundamental framework for quantifying baseline drift and TVSPs from a measured pulse signal in the future.

## 1. Introduction

Arterial pulse signals carry significant cardiovascular (CV) physiological and pathological implications [1,2,3,4]. To date, various arterial indices are derived from virtually every feature of a measured pulse signal for assessing the CV system [1,2,3,4]. For instance, both pulse peak and pulse foot are utilized to calculate pulse transit time (PTT) and pulse wave velocity [5,6]. The features of the arterial pulse waveform (APW) and its first-order and second-order time derivatives are combined in various ways to serve as arterial indices [1,3,7]. A pulse signal is a collection of multiple harmonics of the heart rate, and harmonic information derived from a measured pulse signal also reveals the CV condition [4]. Heart rate variability (HRV), namely, the time duration variations between pulse cycles, is indicative of the CV condition [8].

Although medical instruments (i.e., tonometry and ultrasound) have been used for pulse measurements in numerous clinical studies over the past several decades, their high cost and operational complexity render them impractical for routine clinical utilities and at-home use [1,2,3]. Thus, various sensors have been explored as an affordable, easy-to-use alternative to medical instruments [1,2,3]. These sensors can be categorized into three types: accelerometers, photoplethysmography (PPG) sensors, and tactile sensors [9]. Yet, as with medical instruments, arterial pulse signals measured by these sensors are distorted by motion artifacts (MAs), namely, body motion, respiration, and finger jittering [10,11,12,13,14]. Regardless of sensor type, a sensor needs to be pressed against an artery to establish tissue–sensor contact at the skin surface. Then, the true pulse signal in an artery goes through the transmission path of tissue, tissue–sensor contact, and the sensor, namely, tissue–contact–sensor (TCS) stack, and is recorded as the sensor output (i.e., a measured pulse signal). In pulse measurement, a sensor is either manually held or fixed at an artery. MA alters the distance of the sensor relative to the artery, which is commonly referred to as baseline drift and is channeled into a measured pulse signal. To date, MA is thought to manifest solely as baseline drift in a measured pulse signal [10,11,12,13,14]. Yet, this distance change further alters the dynamic behavior of the transmission path, which is also channeled into the measured pulse signal [9,15].

Given the unavoidable nature of MAs in pulse measurement, numerous studies have focused on developing various data-processing algorithms to estimate and subtract baseline drift from a measured pulse signal to obtain the pulse signal free of MAs, commonly referred to as APW [1,10,11]. This APW is further utilized to derive arterial indices from its features. A large body of literature exists on the working principles of these algorithms and their applications to the measured pulse signals [10,11]. Since MA represents low-frequency signals (<0.7 Hz) relative to a pulse signal (>1 Hz) [16], a data-processing algorithm usually includes a frequency-dependent filtering or decomposition technique and cubic spline estimation (CSE) to estimate and remove baseline drift [11]. Overall, it has been found that different algorithms applied to the same measured pulse signal yield different estimations of baseline drift and result in different APWs [10]. To examine the effectiveness of a data-processing algorithm on estimating baseline drift from a measured pulse signal, the adopted approach is to add assumed baseline drift in different shapes to an ideal pulse signal (i.e., identical pulse cycles) to create a measured pulse signal with the known baseline drift, and then the data-processing algorithm is applied on this signal to extract its baseline drift and compare it with the known one [11]. To the best of the authors’ knowledge, MA is directly equated with baseline drift in all the existing studies on MA estimation, and how the dynamic behavior alteration of the transmission path by MA could possibly affect a measured pulse signal is neglected [10,11]. The neglect of the latter is due to the fact that all the existing studies on MA estimation only account for MA-caused time-varying length changes in the transmission path as baseline drift but neglect the dynamic behavior involvement of the transmission path in pulse measurement.

Recently, a 1DOF (degree-of-freedom) system [9] was created to fully account for the involvement of the transmission path in pulse measurement. This 1DOF system provides a theoretical basis for interpreting variability in measured pulse signals in experimental studies, due to measurement variations in individual (i.e., tissue), contact pressure *P_c_*, and sensor alignment. Based on this 1DOF system, MA channeled into a measured pulse signal can be quantified as (1) a time-varying distance of the sensor relative to the artery, which is exactly the aforementioned baseline drift, and (2) time-varying system parameters (TVSPs) of the 1DOF system, which arises from the dynamic behavior alteration of the transmission path by baseline drift. Baseline drift is well established as additive noise to a measured pulse signal. Yet, how TVSPs affect a measured pulse signal has never been explored to date. In this work, we aim to develop an analytical model of MA that takes fully into account the involvement of the transmission path in pulse measurement and mathematically relates baseline drift and TVSPs to a measured pulse signal. As Part I of the work on this topic, this paper focuses on pulse measurement by accelerometers and PPG sensors. Part II focuses on pulse measurement by tactile sensors.

## 2. Materials and Methods

The analytical model of MA in a measured pulse signal is based on three assumptions: (1) the transmission path from the true pulse signal to the measured pulse signal behaves linearly, and thus, the TCS stack is modeled as a 1DOF system; (2) the TVSPs of the 1DOF system vary linearly with baseline drift; and (3) the true pulse signal in an artery is unaffected in pulse measurement. Given the great utilities of APW in clinical studies and the high sensitivity of APW to MA, we only consider pulse measurement at rest, and thus, MA in this study is <0.7 Hz [16]. Since it is not involved in the transmission path, electrical transduction and the microstructure in an accelerometer are excluded, and the accelerometer and the printed circuit board (PCB) it sits on for pulse measurement are treated as a mass [9]. The transmission path affects optical transduction in a PPG sensor. For simplicity, this effect is considered in terms of displacement here and will be further explained in Section 4. As such, a measured pulse signal is the displacement output of a sensor. Arterial wall displacement and pulsatile pressure in an artery should vary simultaneously in pulse measurement, based on pulse wave theory [4,17]. Hence, we examine the influence of MAs on a measured pulse signal, with the arterial wall displacement and pulsatile pressure as the true pulse signal considered separately for a more complete understanding.

### 2.1. Arterial Wall Displacement as the True Pulse Signal

**Figure 1:**
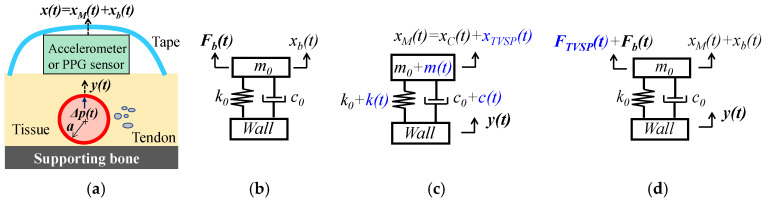
Schematics with arterial wall displacement *y*(*t*) as true pulse signal in an artery: (**a**) pulse measurement with an accelerometer or a PPG sensor fixed via tape. (**b**) 1DOF system of the TCS stack with *x_b_*(*t*) at the mass. (**c**) 1DOF system of the TCS stack containing TVSPs. (**d**) equivalent forces for MA acting on the 1DOF system without TVSPs (Note: TVSP-related terms are in blue).

As shown in Figure 1a,b, an accelerometer or a PPG sensor is fixed above an artery by tape. By exerting *P_c_*, the fixing establishes tissue–sensor contact and forms the TCS stack, which is modeled as a 1DOF system with the sensor being part of the mass [9]. The fixing presets the nominal values of the 1DOF system parameters as $m_{0}$, $k_{0}$, and $c_{0}$. While $\;k_{0}$ and $c_{0}$ are from the tissue, due to its deformability, $m_{0}$ includes the contributions from the tissue and the sensor. As the true pulse signal, arterial wall displacement $y\left(t\right)$ is time-harmonic and serves as the base excitation for the 1DOF system and is time-harmonic [4]:

(1) $$y\left(t\right)=y_{0}e^{j{(\omega }_{y}t+\varphi_{y})}$$

where $y_{0}$, $\varphi_{y}$, and $\omega_{y}$ are the amplitude, phase, and angular frequency of $y\left(t\right)$, respectively.

#### 2.1.1. MA as Baseline Drift and TVSP

As shown in Figure 1b, MA causes a time-varying displacement $x_{b}\left(t\right)$ at the mass:

(2) $$x_{b}\left(t\right)=x_{b0}e^{j{(\omega }_{b}t+\varphi_{b})}$$

where $x_{b0}$, $\varphi_{b}$, and $\omega_{b}$ are the amplitude, phase, and angular frequency of $x_{b}\left(t\right)$, respectively. This displacement is associated with force $F_{b}\left(t\right)$ arising from MA [9]:

(3) $$F_{b}\left(t\right)=m_{0}\cdot \frac{d^{2}x_{b}\left(t\right)}{{dt}^{2}}+c_{0}\cdot \frac{dx_{b}\left(t\right)}{dt}+k_{0}\cdot x_{b}\left(t\right)$$

By altering the TCS stack length, $x_{b}\left(t\right)$ leads to changes in the values of its system parameters, due to its effect on prestress in the tissue [9,15]:

(4) $$m=m_{0}+m\left(t\right),\;c=c_{0}+c\left(t\right),\;k=k_{0}+k\left(t\right)\mathrm{with}\;m\left(t\right),k\left(t\right),c\left(t\right)\propto x_{b}\left(t\right)$$

where $m\left(t\right),k\left(t\right)$, and $c\left(t\right)$ are TVSPs. Since TVSPs are caused by $x_{b}\left(t\right)$, they all have a low frequency (<0.7 Hz).

As shown in Figure 1c, $y\left(t\right)$, as the base excitation, causes displacement $x_{M}\left(t\right)$ at the mass [9]:

(5) $$\left[m_{0}+m\left(t\right)\right]\cdot \frac{d^{2}x_{M}\left(t\right)}{{dt}^{2}}+\left[c_{0}+c\left(t\right)\right]\cdot \frac{dx_{M}\left(t\right)}{dt}+\left[k_{0}+k\left(t\right)\right]\cdot x_{M}\left(t\right)=\left[k_{0}+k\left(t\right)\right]\cdot y\left(t\right)+\left[c_{0}+c\left(t\right)\right]\cdot \frac{dy\left(t\right)}{dt}$$

Due to TVSPs in Equation (5), $x_{M}\left(t\right)$ takes the form [18]:

(6) $${x_{M}\left(t\right)=x}_{T}\left(t\right)\cdot e^{j\varphi_{T}\left(t\right)}\;\mathrm{with}\;\omega_{T}\left(t\right)=\frac{{d\varphi }_{T}\left(t\right)}{dt}$$

where $x_{T}$, $\varphi_{T}$, and $\omega_{T}$ are the instant amplitude, phase, and frequency of $x_{M}\left(t\right)$, respectively. To better reveal the influence of MA, the displacement at the mass is used to represent the output of an accelerometer, instead of acceleration. The measured pulse signal by an accelerometer becomes:

(7a) $$x_{ACC}\left(t\right)=x_{M}\left(t\right)+x_{b}\left(t\right)\;\mathrm{with}\;x_{M}\left(t\right)=x_{TVSP}\left(t\right)+x_{C}\left(t\right)\;\text{(Accelerometer)}$$

where $x_{C}\left(t\right)$ is the displacement at the mass when free of MA (i.e., free of TVSP), and $x_{TVSP}\left(t\right)$ is the TVSP-generated distortion in Equation (5). A PPG sensor monitors the displacement of the arterial wall, and its measured pulse signal is:

(7b) $$x_{PPG}\left(t\right)=y\left(t\right)+g\left(x_{TVSP}\left(t\right)+x_{b}\left(t\right)\right)\;\;\;\;\;\;\;\;\;\;\;\;\;\;\;\;\;\;\;\;\;\;\;\;\;\;\;\;\;\;\text{(PPG sensor)}$$

where $g\left(x_{TVSP}\left(t\right)+x_{b}\left(t\right)\right)$, as a function of $x_{TVSP}\left(t\right)+x_{b}\left(t\right)$, is added due to optical transduction [16].

Based on Equation (5), when free of MA (i.e., free of TVSP), $x_{C}\left(t\right)$ at the mass is [9]:

(8a) $$x_{C}\left(t\right)=x_{0}{\cdot e}^{j{(\omega }_{x}t+\varphi_{x})}=G_{0}e^{j\varphi_{0}}{\cdot y}_{0}e^{j{(\omega }_{y}t+\varphi_{y})}$$

(8b) $$\mathrm{with}\;G_{0}e^{j\varphi_{0}}=\frac{k_{0}+c_{0}j\omega_{y}}{-{m_{0}\omega }_{y}^{2}+c_{0}\omega_{y}j+k_{0}};\;\omega_{x}=\omega_{y};\;\varphi_{x}=\varphi_{y}+\varphi_{0};\;x_{0}=G_{0}{\cdot y}_{0}$$

where $x_{0}$, $\varphi_{x}$, and $\omega_{x}$ are the amplitude, phase, and frequency of $x_{C}\left(t\right)$, respectively. Consequently, the measured pulse signals free of MA by an accelerometer and a PPG sensor are $x_{C}\left(t\right)$ and *y*(*t*), respectively. TVSPs alter the measured pulse signal at the mass by $x_{TVSP}\left(t\right)$, and the total distortion caused by MA in an accelerometer measurement is:

(9a) $$x_{ACC-MA}\left(t\right)=x_{TVSP}\left(t\right)+x_{b}\left(t\right)\;\mathrm{with}\;x_{TVSP}\left(t\right)=x_{M}\left(t\right)-x_{C}\left(t\right)\;\text{(Accelerometer)}$$

Similarly, total distortion caused by MA in a PPG sensor measurement becomes:

(9b) $$x_{PPG-MA}\left(t\right)=g\left(x_{TVSP}\left(t\right)+x_{b}\left(t\right)\right)\;\text{(PPG sensor)}$$

#### 2.1.2. MA as Equivalent Forces

To gain a better understanding of the influence of MA on a measured pulse signal, we keep the 1DOF system parameters unaltered, and the influence of MA is accounted for by equivalent forces acting on the sensor, as shown in Figure 1d. Then, the 1DOF system is subject to two inputs, base excitation $y\left(t\right)$ and $F_{MA}\left(t\right)$ on the mass:

(10) $$m_{0}\cdot \frac{d^{2}x\left(t\right)}{{dt}^{2}}+c_{0}\cdot \frac{dx\left(t\right)}{dt}+k_{0}\cdot x\left(t\right)=k_{0}\cdot y\left(t\right)+c_{0}\cdot \frac{dy\left(t\right)}{dt}+F_{MA}\left(t\right)$$

Together with $y\left(t\right)$, $F_{MA}\left(t\right)$ should lead to the same displacement in Equation (7a). Comparison of Equations (3), (6), and (8) gives rise to $F_{MA}\left(t\right)$:

(11a) $$F_{MA}\left(t\right)=F_{TVSP}\left(t\right)+F_{b}\left(t\right)\;\mathrm{with}\;F_{TVSP}\left(t\right)=F_{T}{\left(t\right)-F}_{C}\left(t\right)$$

where

(11b) $$F_{T}\left(t\right)=m_{0}{\cdot \left(x_{T}\left(t\right)e^{j\varphi_{T}\left(t\right)}\right)}^{\prime \prime }+c_{0}\cdot {\left(x_{T}\left(t\right)e^{j\varphi_{T}\left(t\right)}\right)}^{\prime }+k_{0}{\cdot x}_{T}\left(t\right)e^{j\varphi_{T}\left(t\right)}$$

(11c) $$F_{C}\left(t\right)=k_{0}\cdot y\left(t\right)+c_{0}\cdot \frac{dy\left(t\right)}{dt}$$

Note that $F_{C}\left(t\right)$ is the equivalent force acting on the mass from the base excitation $y\left(t\right)$. As such, the influence of MA on a measured pulse signal can be accounted for by the sum of $F_{b}\left(t\right)$ and $F_{TVSP}\left(t\right)$ acting on the sensor.

### 2.2. Pulsatile Pressure as the True Pulse Signal

**Figure 2:**
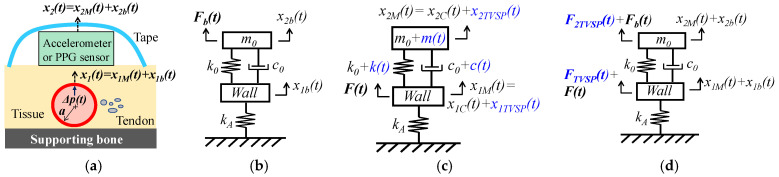
Schematics with pulsatile pressure *Δp*(*t*) as true pulse signal in an artery: (**a**) pulse measurement with an accelerometer and a PPG sensor fixed via tape. (**b**) 2DOF system of the TCS stack and arterial wall with *x_2b_*(*t*) at the mass. (**c**) 2DOF system with the TCS stack containing TVSPs. (**d**) equivalent forces for MA acting on the 2DOF system without TVSPs (Note: TVSP-related terms are in blue).

Since the inertia and damping terms of the arterial wall are negligible [1,2,3,4,9], relative to its elastic term, the arterial wall is modeled as a spring with its stiffness *k_A_*, as shown in Figure 2a,b. While one end of the spring is fixed, its other end is connected to the TCS stack. Adding the arterial wall to the TCS stack forms a 2DOF system. The pulsatile pressure $\Delta p\left(t\right)$ is considered as the true pulse signal and translates to a force $F\left(t\right)$ acting on the arterial wall [4,9]:

(12) $$F\left(t\right)=F_{0}e^{j{(\omega }_{p}t+\varphi_{p})}=\pi a\Delta p\left(t\right)\;\;\;\;\mathrm{with}\;\Delta p\left(t\right)={\Delta p}_{0}e^{j{(\omega }_{p}t+\varphi_{p})}$$

where $F_{0}$, $\varphi_{p}$, and $\omega_{p}$ are the amplitude, phase, and angular frequency of $F\left(t\right)$, respectively.

#### 2.2.1. MA as Baseline Drift and TVSP

As shown in Figure 2b, MA causes a time-varying displacement $x_{2b}\left(t\right)$ at the mass:

(13) $$x_{2b}\left(t\right)=x_{2b0}e^{j{(\omega }_{2b}t+\varphi_{2b})}$$

It leads to a time-varying displacement $x_{1b}\left(t\right)$ at the arterial wall. Based on force balance, $x_{2b}\left(t\right)$ and $x_{1b}\left(t\right)$ are related by [9]:

(14a) $$\left(k_{A}+k_{0}\right)\cdot x_{1b}\left(t\right)-{k_{0}\cdot x}_{2b}\left(t\right)+c_{0}\cdot \left[\frac{{dx}_{1b}\left(t\right)}{dt}-\frac{{dx}_{2b}\left(t\right)}{dt}\right]=0$$

(14b) $$k_{0}\cdot \left[x_{2b}\left(t\right)-x_{1b}\left(t\right)\right]+c_{0}\cdot \left[\frac{{dx}_{2b}\left(t\right)}{dt}-\frac{{dx}_{1b}\left(t\right)}{dt}\right]+m_{0}\cdot \frac{{d^{2}x}_{2b}\left(t\right)}{{dt}^{2}}=F_{b}\left(t\right)$$

where $F_{b}\left(t\right)$ stems from MA. The TVSPs of the 1DOF system in Equation (4) are functions of $x_{2b}\left(t\right)-x_{1b}\left(t\right)$:

(15) $$k\left(t\right),c\left(t\right),m\left(t\right)\propto x_{2b}\left(t\right)-x_{1b}\left(t\right)$$

As shown in Figure 2c, in response to $F\left(t\right)$, the displacements at the mass and the wall are governed by:

(16a) $$\left(k_{A}+k_{0}+k\left(t\right)\right){\cdot x}_{1M}\left(t\right)-\left(k_{0}+k\left(t\right)\right)\cdot x_{2M}\left(t\right)+\left(c_{0}+c\left(t\right)\right)\cdot \left[\frac{{dx}_{1M}\left(t\right)}{dt}-\frac{{dx}_{2M}\left(t\right)}{dt}\right]=F\left(t\right)$$

(16b) $$\left(k_{0}+k\left(t\right)\right)\cdot \left[x_{2M}\left(t\right)-x_{1M}\left(t\right)\right]+\left(c_{0}+c\left(t\right)\right)\cdot \left[\frac{{dx}_{2M}\left(t\right)}{dt}-\frac{{dx}_{1M}\left(t\right)}{dt}\right]+\left(m_{0}+m\left(t\right)\right)\cdot \frac{{d^{2}x}_{2M}\left(t\right)}{{dt}^{2}}=0$$

The solutions to $x_{1M}\left(t\right)$ and $x_{2M}\left(t\right)$ take the forms [18]:

(17a) $$x_{1M}\left(t\right)=x_{1T}{\left(t\right)e}^{j\varphi_{1T}\left(t\right)}\;\mathrm{with}\;\omega_{1T}\left(t\right)=\frac{{d\varphi }_{1T}\left(t\right)}{dt}$$

(17b) $$x_{2M}\left(t\right)=x_{2T}{\left(t\right)e}^{j\varphi_{2T}\left(t\right)}\;\mathrm{with}\;\omega_{2T}\left(t\right)=\frac{{d\varphi }_{T2}\left(t\right)}{dt}$$

where $x_{1T}$, $\varphi_{1T}$, and $\omega_{1T}$ are the instant amplitude, phase, and frequency of $x_{1M}\left(t\right)$, respectively, and $x_{2T}$, $\varphi_{2T}$, and $\varphi_{2T}$ are the instant amplitude, phase, and frequency of $x_{2M}\left(t\right)$, respectively. The measured pulse signals by an accelerometer $x_{ACC}\left(t\right)$ and a PPG sensor $x_{PPG}\left(t\right)$ become:

(18a) $$x_{ACC}\left(t\right)=x_{2M}\left(t\right)+x_{2b}\left(t\right)\mathrm{with}\;x_{2M}\left(t\right)=x_{2C}\left(t\right){+x}_{2TVSP}\left(t\right)\;\;\text{(accelerometer)}$$

(18b) $$x_{PPG}\left(t\right)=x_{1M}\left(t\right)+x_{1b}\left(t\right)+g\left(x_{2TVSP}\left(t\right)+x_{2b}\left(t\right)\right)\mathrm{with}\;x_{1M}\left(t\right)=x_{1C}\left(t\right){+x}_{1TVSP}\left(t\right)\;\text{(PPG sensor)}$$

where $x_{2C}\left(t\right)$ and $x_{1C}\left(t\right)$ are the displacement at the mass and the wall, respectively, when free of MA (i.e., free of TVSP), and *x*_2*TVSP*_(*t*) and *x*_1*TVSP*_(*t*) are the TVSP-generated distortion at the mass and the wall, respectively, in Equation (16). Note that $g\left(x_{2TVSP}\left(t\right)+x_{2b}\left(t\right)\right)$ in Equation (18b) is due to optical transduction, which will be explained in Section 4.

Based on Equation (16), when free of MA, the displacements $x_{1C}\left(t\right)$ and $x_{2C}\left(t\right)$ at the arterial wall and the mass, respectively, are related to $F\left(t\right)$ by [9]:

(19a) $$x_{1C}\left(t\right)=x_{10}e^{j{(\omega }_{1x}t+\varphi_{1x})}=G_{10}e^{j\varphi_{10}}F_{0}e^{j{(\omega }_{p}t+\varphi_{p})}$$

(19b) $$\mathrm{with}\;G_{10}e^{j\varphi_{10}}=\frac{1}{k_{A}-\frac{\left\{{c_{0}\omega }_{p}j+k_{0}\right\}m_{0}\omega_{p}^{2}}{c_{0}\omega_{p}j+k_{0}-m_{0}\omega_{p}^{2}}};\;\omega_{1x}=\omega_{p};\;\varphi_{1x}=\varphi_{p}+\varphi_{10};\;x_{10}=G_{10}F_{0}$$

(19c) $$x_{2C}\left(t\right)=x_{20}e^{j{(\omega }_{2x}t+\varphi_{2x})}=G_{20}e^{j\varphi_{20}}F_{0}e^{j{(\omega }_{p}t+\varphi_{p})}$$

(19d) $$\mathrm{with}\;G_{20}e^{j\varphi_{20}}=\frac{1}{k_{A}\left\{\frac{-m_{0}\omega_{p}^{2}}{c_{0}\omega_{p}j+k_{0}}+1\right\}-m_{0}\omega_{p}^{2}};\;\omega_{2x}=\omega_{p};\;\varphi_{2x}=\varphi_{p}+\varphi_{20};\;{\;x}_{20}=G_{20}F_{0}$$

Consequently, the measured pulse signals by an accelerometer and a PPG sensor become $x_{2C}\left(t\right)$ and $x_{1C}\left(t\right)$, respectively. The total distortions from MA in an accelerometer measurement $x_{ACC-MA}\left(t\right)$ and in a PPG sensor measurement $x_{PPG-MA}\left(t\right)$ become:

(20a) $$x_{ACC-MA}\left(t\right)=x_{2TVSP}\left(t\right)+x_{2b}\left(t\right)\mathrm{with}\;x_{2TVSP}\left(t\right)=x_{2M}\left(t\right)-x_{2C}\left(t\right)\;\text{(accelerometer)}$$

(20b) $$x_{PPG-MA}\left(t\right)=x_{1TVSP}\left(t\right)+x_{1b}\left(t\right)+g\left(x_{2TVSP}\left(t\right)+x_{2b}\left(t\right)\right)\mathrm{with}\;x_{1TVSP}\left(t\right)=x_{1M}\left(t\right)-x_{1C}\left(t\right)\;\text{(PPG sensor)}$$

#### 2.2.2. MA as Equivalent Forces

With the 1DOF system of the TCS stack unaltered, as shown in Figure 2d, we derive equivalent forces for MA, which provide the displacement at the mass and the wall:

(21a) $$x_{2}\left(t\right)=x_{2M}\left(t\right)+x_{2b}\left(t\right)-x_{2C}\left(t\right)$$

(21b) $$x_{1}\left(t\right)=x_{1M}\left(t\right)+x_{1b}\left(t\right)-x_{1C}\left(t\right)$$

The equivalent force for $x_{1b}\left(t\right)$ and $x_{2b}\left(t\right)$ is $F_{b}\left(t\right)$ in Equation (14). Based on Equation (16), an equivalent force for $x_{1C}\left(t\right)$ and $x_{2C}\left(t\right)$ should be −F(t) acting on the arterial wall. Equivalent forces for $x_{1M}\left(t\right)$ and $x_{2M}\left(t\right)$ are given by:

(22a) $$\left(k_{A}+k_{0}\right)\cdot x_{1M}\left(t\right)-k_{0}x_{2M}\left(t\right)+c_{0}\cdot \left[\frac{{dx}_{1M}\left(t\right)}{dt}-\frac{{dx}_{2M}\left(t\right)}{dt}\right]=F_{1T}\left(t\right)$$

(22b) $${\;\;k}_{0}\cdot \left[x_{2M}\left(t\right)-x_{1M}\left(t\right)\right]+c_{0}\cdot \left[\frac{{dx}_{2M}\left(t\right)}{dt}-\frac{{dx}_{1M}\left(t\right)}{dt}\right]+m_{0}\frac{{d^{2}x}_{2M}\left(t\right)}{{dt}^{2}}=F_{2T}\left(t\right)$$

Taken together, the influence of MA on a measured pulse signal can be accounted for by equivalent forces $F_{1MA}\left(t\right)$ acting on the arterial wall and $F_{2MA}\left(t\right)$ acting on the sensor:

(23a) $$F_{1MA}\left(t\right)=F_{1TVSP}\left(t\right)=F_{1T}\left(t\right)-F\left(t\right)$$

(23b) $${F_{2MA}\left(t\right)=F_{2TVSP}\left(t\right)+F}_{b}\left(t\right)\;\mathrm{with}\;F_{2TVSP}\left(t\right)=F_{2T}\left(t\right)$$

As such, it is the combination of $F_{1TVSP}\left(t\right)$ acting on the arterial wall and ${F_{2TVSP}\left(t\right)+F}_{b}\left(t\right)$ acting on the sensor that leads to the distortion in a measured pulse signal in Equation (20). Note that $F_{1TVSP}\left(t\right)$ and $F_{2TVSP}\left(t\right)$ are not time-harmonic, similar to $x_{1TVSP}\left(t\right)$ and $x_{2TVSP}\left(t\right)$ in Equation (17).

### 2.3. Numerical Calculation

The pulse signals at the carotid artery (CA) for healthy 25-year-old virtual subjects [19] are chosen for analysis. The nominal values for the 1DOF system parameters are chosen from [9]: $k_{0}=1/6\cdot k_{A}$, $r_{0}=\omega_{0}/\omega_{C}=\;2$, and $\zeta_{0}=\;1.5$, where $\omega_{0\;}$ and $\zeta_{0}$ are the nominal frequency and damping factor of the 1DOF system, with $\omega_{C}$ being the frequency of the heart rate (HR). Baseline drift $x_{b}(t)$ in Figure 1 and baseline drift $x_{2b}(t)$ in Figure 2 are from the CSE-estimated baseline drift from a measured pulse signal (see Figure 18 in Part II). Owing to the geometrical and anatomical complexity surrounding an artery, no direct mathematical, linear relation can be expected between baseline drift and TVSPs. To clarify their collective manifestation in a measured pulse signal, we assume that the TVSPs hold a linear relation with baseline drift in Figure 1 [9]:

(24) $$k\left(t\right)=-\frac{k_{0}}{3}\cdot \frac{x_{b}\left(t\right)}{\max\left(x_{b}\left(t\right)\right)};\;c\left(t\right)=-\frac{c_{0}}{3}\cdot \frac{x_{b}\left(t\right)}{\max\left(x_{b}\left(t\right)\right)};\;m\left(t\right)=-\frac{m_{0}}{2}\cdot \frac{x_{b}\left(t\right)}{\max\left(x_{b}\left(t\right)\right)}$$

Note that m(t) varies faster with baseline drift than k(t) for reduced natural frequency with baseline drift [9,15]. Similarly, the TVSPs in Figure 2 hold the above relations with $x_{2b}(t)-x_{1b}(t)$. Based on these parameters, measured pulse signals can be calculated using the analytical model presented here.

All the calculations are conducted in MATLAB2024a. The built-in function ODE45 is utilized for all the time-domain calculations. After the measured pulse signals and the equivalent forces for MA are obtained, Fast Fourier transform (FFT) is applied to them for their frequency spectrum. The contributions of the baseline drift and TVSPs to a measured pulse signal are separated in the time domain and frequency domain for their respective manifestations in a measured pulse signal.

## 3. Results

### 3.1. Arterial Wall Displacement as the True Pulse Signal

**Figure 3:**
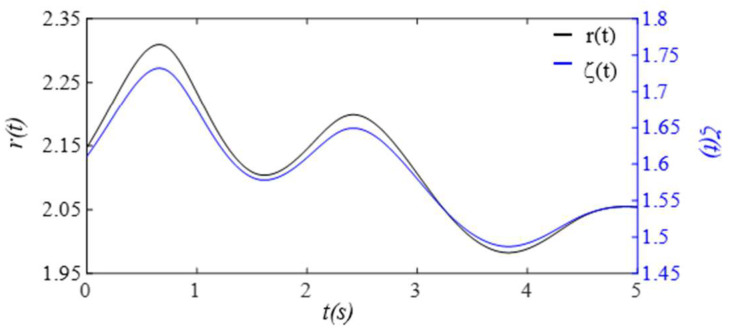
Frequency ratio *r*(*t*) and damping factor *ζ*(*t*).

**Figure 4:**
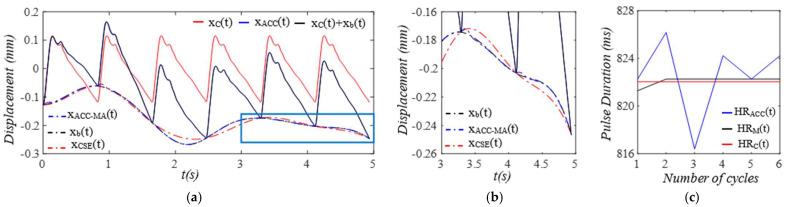
Calculated measurement at the mass (or accelerometer measurement). (**a**) pulse signals: *x_C_*(*t*), *x_ACC_*(*t*), and *x_C_*(*t*) + *x_b_*(*t*); MA−related signals: *x_b_*(*t*), *x_ACC__−MA_*(*t*), and *x_CSE_*(*t*). (**b**) zoomed−in view of MA−related signals. (**c**) HR: *HR_ACC_*(*t*), *HR_M_*(*t*), and *HR_C_*(*t*).

Given the clear implications of the frequency ratio $r(t)\;=\omega (t)/\omega_{C}$ and damping factor ζ(t)  of the 1DOF system to its dynamic behavior, Figure 3 shows these two parameters for the effect of the TVSPs on the parameters of the TCS stack. As shown in Figure 4a, baseline drift $x_{b}(t)$ is relatively smooth and large in the measured pulse signal $x_{ACC}(t)$. Note that $x_{C}(t)+x_{b}(t)$ is the pulse signal excluding $x_{TVSP}\left(t\right)$. Although $x_{TVSP}\left(t\right)$ in $x_{ACC}(t)$ is invisible in Figure 4a, as shown in Figure 4b, due to $x_{TVSP}\left(t\right)$, $x_{ACC-MA}\left(t\right)=\;x_{b}(t)+\;x_{TVSP}\left(t\right)$ becomes a non-smooth curve with abrupt changes, relative to $x_{b}(t)$. The CSE-estimated baseline drift $x_{CSE}(t)$ (no filtering is used before it to avoid any unintended distortion) fails to match $x_{b}(t)$. In Figure 4c, *HR_ACC_* and *HR_M_* represent heart rates (HR) derived from $x_{ACC}(t)$ (containing $x_{ACC-MA}\left(t\right)$) and $x_{M}\left(t\right)$ (containing only $x_{TVSP}\left(t\right)$), respectively. *HR_C_* derived from $x_{C}(t)$ is also plotted. Evidently, $x_{TVSP}\left(t\right)$ and $x_{ACC-MA}\left(t\right)$ cause different HR variations between pulse cycles, indicating that $x_{b}(t)$ and $x_{TVSP}\left(t\right)$ both shift the start/end of a pulse cycle.

**Figure 5:**
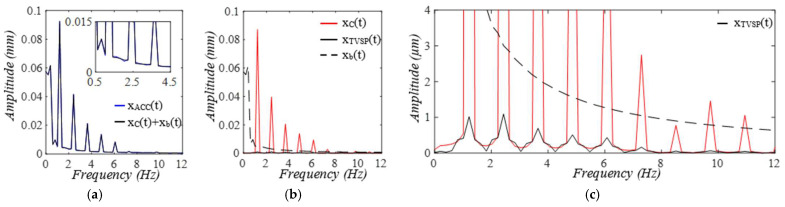
Frequency spectrum of (**a**) *x_ACC_*(*t*)) and *x_C_*(*t*) + *x_b_*(*t*). (**b**) *x_C_*(*t*), *x_TVSP_*(*t*), and *x_b_*(*t*). (**c**) zoomed-in view of *x_TVSP_*(*t*).

As shown in Figure 5, each harmonic of $x_{C}(t)$ appears as a distinct, sharp peak. In contrast, in the frequency spectrum of $x_{ACC}(t)$, while a low-frequency (<1 Hz) peak arises from $x_{b}(t)$, TVSPs generate low-amplitude signals centering around each harmonic of the true pulse signal, due to its multiplication with the true pulse signal. Since r(t) is around 2, the second harmonic corresponds to the natural frequency of the 1DOF system, and thus, its TVSP-generated distortion is amplified relative to the other harmonics.

**Figure 6:**
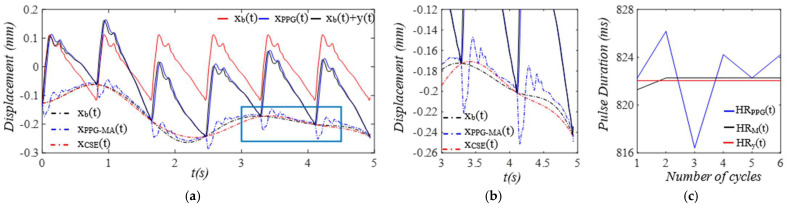
Calculated measurement at the arterial wall (or PPG sensor measurement). (**a**) pulse signals: *y*(*t*), *x_PPG_*(*t*), and *y*(*t*) *+ x_b_*(*t*); MA-related signals: *x_b_*(*t*), *x_PPG__−MA_*(*t*), and *x_CSE_*(*t*). (**b**) zoomed−in view of MA−related signals. (**c**) HR: *HR_PPG_*(*t*), *HR_M_*(*t*), and *HR_y_*(*t*).

**Figure 7:**
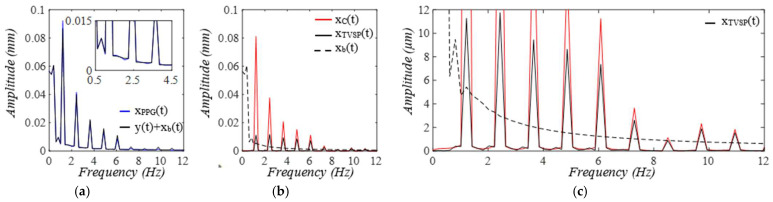
Frequency spectrum of (**a**) *x_PPG_*(*t*), *y*(*t*) *+ x_b_*(*t*). (**b**) *y*(*t*), *x_TVSP_*(*t*), and *x_b_*(*t*). (**c**) zoomed-in view of *x_TVSP_*(*t*).

Optical transduction in the transmission path is rather complicated [16]. For simplicity, we assume that g(*x_TVSP_*(*t*) + *x_b_*(*t*)) = *x_TVSP_*(*t*) + *x_b_*(*t*) in Equation (7b). Then, the total distortion in the measured pulse signal *x_PPG_*(*t*) is *x_PPG-MA_*(*t*) = *x_TVSP_*(*t*) + *x_b_*(*t*). As shown in Figure 6, the measured pulse signal $x_{PPG}\left(t\right)$ is severely distorted by $x_{TVSP}\left(t\right)$, as compared to *x_ACC_*(*t*) in Figure 4. The CSE-estimated baseline drift $x_{CSE}(t)$ fails to match $x_{b}\left(t\right)$. In Figure 6c, *HR_PPG_* and *HR_M_* represent heart rates (HRs) derived from $x_{PPG}(t)$ (containing $x_{PPG-MA}\left(t\right)$) and $y_{M}(t)=y\left(t\right)+x_{TVSP}\left(t\right)$ (containing only $x_{TVSP}\left(t\right)$), respectively. *HR_y_* derived from y(t) is also plotted. Evidently, $x_{TVSP}\left(t\right)$ and $x_{PPG-MA}\left(t\right)$ cause different HR variations between pulse cycles. Note that *HR_PPG_* and *HR_M_* in Figure 6c are very close to their counterparts in Figure 4c. As shown in Figure 7, each harmonic of *y*(*t*) appears as a distinct, sharp peak. Baseline drift *x_b_*(*t*) is identical to that in Figure 5. However, in Figure 7c, the large $x_{TVSP}\left(t\right)$ in *x_PPG_*(*t*) leads to relatively large low-amplitude signals centering around each harmonic, as compared to its counterparts in Figure 5c.

**Figure 8:**
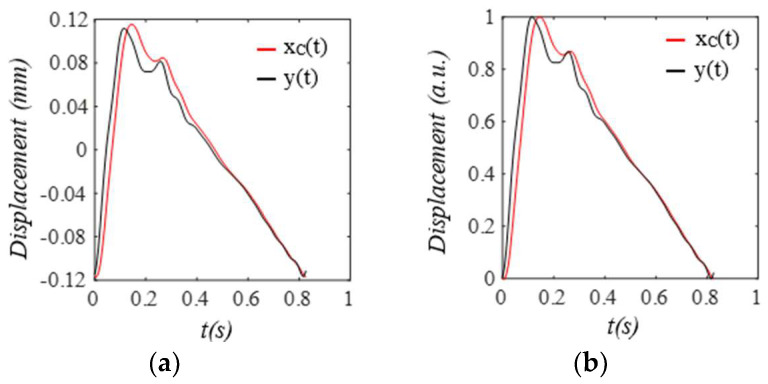
The amplitude and APW of *x_C_*(*t*) are different from the true pulse signal *y*(*t*), due to the harmonic−dependent transfer function of the TCS stack. (**a**) pulse signals. (**b**) their normalized APWs.

As shown in Figure 8, compared to the true pulse signal y(t), the amplitude of $x_{C}(t)$ is slightly increased by the TCS stack, and its waveform is altered, with a noticeable phase delay relative to y(t). This stems from the harmonic-dependent transfer function of the TCS stack between the two signals, as shown in Equation (8). This phase delay explains (1) why *x_TVSP_*(*t*) in *x_PPG_*(*t*) is larger than that in *x_ACC_*(*t*), because *x_TVSP_*(*t*) rides on *x_C_*(*t*); and (2) why there is great similarity between Figure 4c and Figure 6c, because of the same phase delay between *x_C_*(*t*) and *y*(*t*) in their pulse cycles.

**Figure 9:**
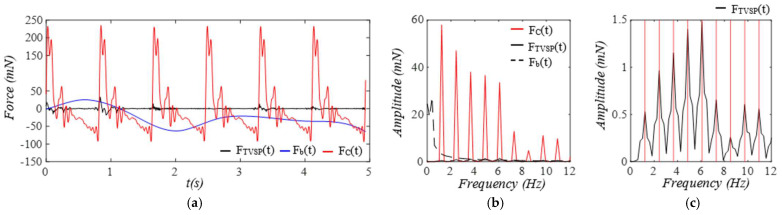
Calculated equivalent forces for MA. (**a**) *F_TVSP_*(*t*), *F_b_*(*t*), and *F_C_*(*t*). (**b**) frequency spectrum of *F_C_*(*t*), *F_TVSP_*(*t*), and *F_b_*(*t*) and (**c**) frequency spectrum of *F_TVSP_*(*t*).

As shown in Figure 9, as compared to equivalent force $F_{C}(t)$ from y(t), equivalent forces for MA are relatively small. While $F_{b}(t)$ has a low-frequency peak, $F_{TVSP}(t)$ has its frequencies centering around each harmonic of the true pulse signal.

### 3.2. Pulsatile Pressure as the True Pulse Signal

**Figure 10:**
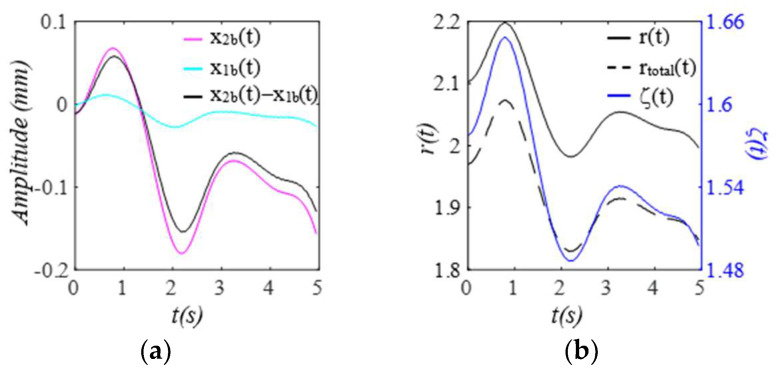
Baseline drifts and its effect on the 1DOF system of the TCS stack: (**a**) *x*_2*b*_(*t*), *x*_1*b*_(*t*), and *x*_2*b*_(*t*)*−x*_1*b*_(*t*). (**b**) *r*(*t*), *r_total_*(*t*), and *ζ*(*t*).

**Figure 11:**
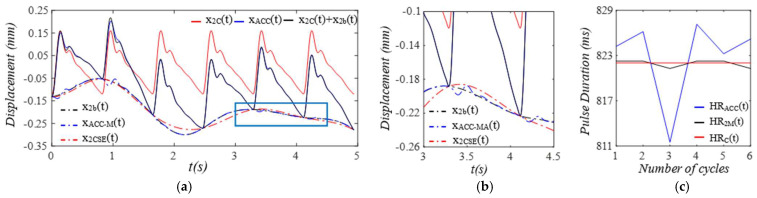
Calculated measurement at the mass (or accelerometer measurement). (**a**) pulse signals: *x_2C_*(*t*), *x_ACC_*(*t*), and *x_2C_*(*t*) *+ x_2b_*(*t*); MA−related signals: *x_2b_*(*t*), *x_ACC-MA_*(*t*), and *x_2CSE_*(*t*). (**b**) zoomed−in view of MA−related signals. (**c**) HR: *HR_ACC_*(*t*), *HR_2M_*(*t*), *HR_C_*(*t*).

**Figure 12:**
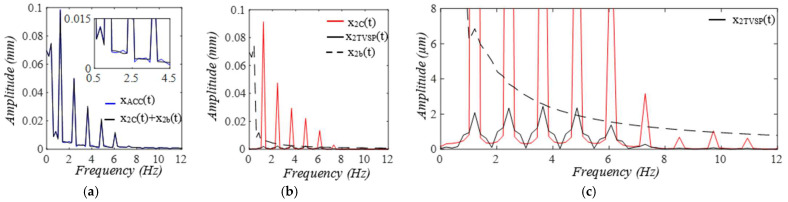
Frequency spectrum of (**a**) *x_ACC_*(*t*), *x_2C_*(*t*) *+ x_2b_*. (**b**) *x_2C_*(*t*), *x_2TVSP_*(*t*), *x_2b_*(*t*). (**c**) zoomed-in view of *x_2TVSP_*(*t*).

When pulsatile pressure Δp(t) (i.e., F(t))  serves as the true pulse signal, as shown in Figure 10, $x_{2b}(t)$ indicates the actual MA, and the arterial wall also encounters baseline drift $x_{1b}(t)$. The changing trends of r(t) and ζ(t)  of the TCS stack follow that of $x_{2b}\left(t\right)-x_{1b}(t)$, which dictates the TVSPs. Note that $r_{total}(t)$ is the frequency ratio accounting for *k_A_*. As shown in Figure 11, TVSPs generate relatively abrupt and small visible distortion in $x_{ACC}(t)$ relative to baseline drift $x_{2b}(t)$. The CSE-estimated baseline drift $x_{2CSE}\left(t\right)$ fails to match $x_{2b}\left(t\right)$. As shown in Figure 11c, $x_{2TVSP}\left(t\right)$ and $x_{2b}\left(t\right)$ each cause their own shifts in the start/end of the pulse cycles. As shown in Figure 12, $x_{2TVSP}\left(t\right)$ has its low-amplitude signals centering around each harmonic of the true pulse signal.

**Figure 13:**
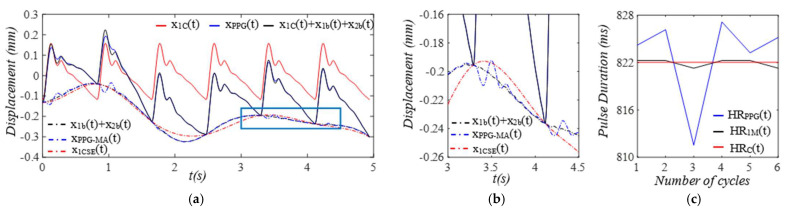
Calculated measurement at the arterial wall (or PPG measurement). (**a**) pulse signals: *x*_1*C*_(*t*), *x_PPG_*(*t*), and *x*_1*C*_(*t*)*+ x*_1*b*_(*t*) *+ x_2b_*(*t*); MA−related signals: *x*_1*b*_(*t*) *+ x_2b_*(*t*), *x_PPG__−MA_*(*t*), and *x*_1*CSE*_(*t*). (**b**) zoomed−in view of MA−related signals. (**c**) HR: *HR_PPG_*(*t*), *HR_1M_*(*t*), and *HR_C_*(*t*).

**Figure 14:**
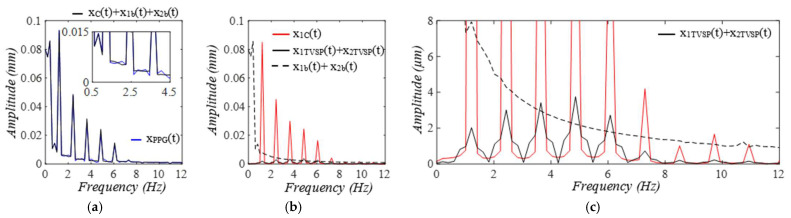
Frequency spectrum of (**a**) x_PPG_ and x_1C_(t) + x_1b_(t) + x_2b_(t). **(b)** x_1C_(t), x_1TVSP_(t) + x_2TVSP_(t), and x_1b_(t)+ x_2b_(t). (**c**) zoomed-in view of x_1TVSP_(t) + x_2TVSP_(t).

**Figure 15:**
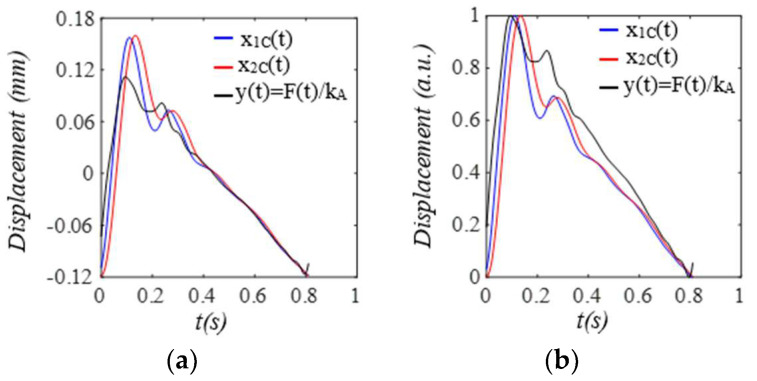
The amplitude and APW of measured pulse signals free of MA: *x*_1*C*_(*t*) and *x*_2*C*_(*t*), are different from the true pulse signal *y*(*t*) *= F*(*t*)*/k_A_* due to the harmonic−dependent transfer function of the TCS stack in Equation (19). (**a**) pulse signals. (**b**) their normalized APWs.

To plot the measured pulse signal by a PPG sensor, we assume that g(*x_2TVSP_*(*t*) *+ x_2b_*(*t*)) = *x_2TVSP_*(*t*) *+ x_2b_*(*t*) in Equation (18b). As shown in Figure 13, $x_{PPG}\left(t\right)$ is noticeably distorted by the TVSPs in the first two pulse cycles, as compared to baseline drift $x_{2b}\left(t\right)+x_{1b}(t)$ in it. Although $x_{2b}\left(t\right)+x_{1b}(t)$ and $x_{PPG-MA}\left(t\right)$ both seem to align well with the start/end of the pulse cycles, the difference between *HR_PPG_* derived from $x_{PPG}\left(t\right)$ and *HR_1M_* derived from $x_{1M}\left(t\right)$ reveals their different shifts in the start/end of the pulse cycles. The CSE-estimated baseline drift $x_{1CSE}\left(t\right)$ fails to match $x_{2b}\left(t\right)+x_{1b}(t)$. As shown in Figure 14, the signals in $x_{1TVSP}\left(t\right)$ centering around the third~fifth harmonics are evidently large, as compared to the rest harmonics. As shown in Figure 15, both the amplitude and APW of the two measured pulse signals free of MA $x_{2C}\left(t\right)$ and $x_{1C}\left(t\right)$ differ greatly from the true pulse signal free of measurement: $y(t)=F(t)/k_{A}$, as shown in Equation (19). However, the difference between $x_{2C}\left(t\right)$ and $x_{1C}\left(t\right)$ is relatively moderate.

**Figure 16:**
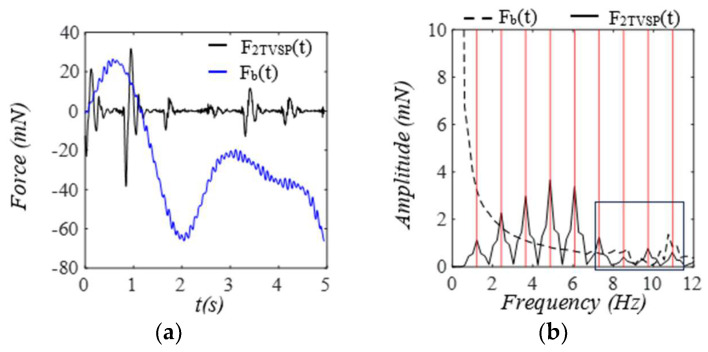
Calculated equivalent forces for MA acting on the mass. (**a**) *F_2TVSP_*(*t*) and *F_b_*(*t*). (**b**) frequency spectrum of *F_2TVSP_*(*t*) and *F_b_*(*t*).

**Figure 17:**
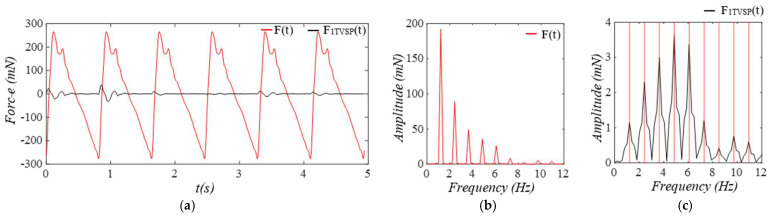
Calculated equivalent forces for MA acting on the arterial wall. (**a**) *F*(*t*) and *F_1TVSP_*(*t*). (**b**) frequency spectrum of *F*(*t*). (**c**) frequency spectrum of *F_1TVSP_*(*t*).

As shown in Figure 16, the equivalent force for the TVSPs $F_{2TVSP}(t)$ on the mass is comparable to $F_{b}$ in magnitude in the first two pulse cycles. The equivalent force $F_{b}\left(t\right)$ for $x_{2b}\left(t\right)$ acting on the mass is mostly in the low frequency range, but it has a small portion at higher harmonics, possibly due to the interaction of the TCS stack and the arterial wall. As shown in Figure 17, $F_{1TVSP}(t)$ on the arterial wall is small relative to F(t). While F(t) has a distinct, sharp peak around its each harmonic, $F_{1TVSP}(t)$ has its signals centering around each harmonic of F(t).

## 4. Discussion

### 4.1. A Full Consideration of the Transmission Path in a Measured Pulse Signal

#### 4.1.1. Comparison with the Current Studies on Pulse Measurement and MA

Owing to MA, a measured pulse signal reveals a non-flat baseline (baseline drift) [1,10,11,12,13,14]. Since MA alters the distance of a sensor relative to the artery over time, intuitively, baseline drift is equated with MA and is deemed as low-frequency additive noise [1,10,11,12,13,14]. As such, various data-processing algorithms, regardless of wavelet-based filtering, Empirical Mode decomposition (EMD), as well as their evolved versions, are built upon the low-frequency nature of baseline drift. These filtering and decomposing techniques are aimed to separate the measured pulse signal into low-frequency components and high-frequency components, with low-frequency components being deemed as baseline drift. Yet, after this stage, the baseline in the filtered pulse signal is usually not a flat line, and CSE is used to estimate a curve for the filtered pulse signal as the extra baseline drift to force it to have a flat baseline [1,10,11]. This pulse signal with a flat baseline is deemed as the APW free of MA. Yet, the true pulse signal itself is a collection of harmonics of the heart rate [4,18]. As a low-frequency signal, this extra baseline drift essentially adds distortion to the obtained APW. Additionally, filtering and EMD may introduce their own distortion to a measured pulse signal [10].

**Figure 18:**
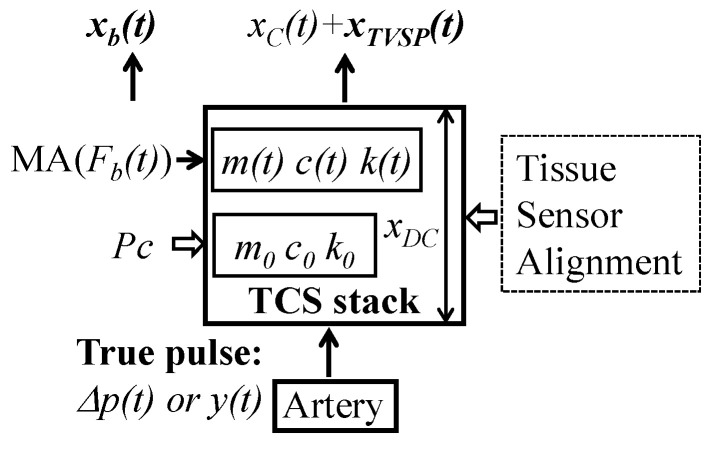
Schematic of all the factors of the transmission path in pulse measurement.

As shown in Figure 18, numerous experimental studies have established that variations in measured pulse signals (i.e., APW and amplitude) lie in the factors of the TCS stack (transmission path): tissue, sensor, alignment, and *P_c_*; when free of MA, *P_c_* is used to establish tissue–sensor contact, during which the TCS stack length $x_{DC}\;$ is fixed and the tissue is prestressed, leading to the nominal values of $m_{0}$, $k_{0}$, and $c_{0}$. The well-known effect of *P_c_* on a measured pulse signal (i.e., APW and amplitude) can be fully accounted for by the values of $m_{0}$, $k_{0}$, and $c_{0}$. The 1DOF system model of the TCS stack provides a theoretical basis for interpreting variations in measured pulse signals [9]. Based on this model, the TCS stack serves as a harmonic-dependent transfer function, and thus, a measured pulse signal always deviates from the true pulse signal.

To date, all the studies on pulse measurement have excluded the TCS stack and treated a measured pulse signal directly as the true pulse signal [1,10,11,12,13,14,16]. Yet, only when MA needs to be included in a measured pulse signal does the TCS stack come in, and MA in a measured pulse signal is solely considered as its length change (baseline drift) [1,10,11,12,13,14,16]. This is understandable, in the sense that treating MA solely as a length change of the TCS stack is consistent with the condition for treating a measured pulse signal as the true pulse signal: the TCS stack is assumed nondeformable and thus can be neglected. As detailed in Section 2, MA causes a length change of the TCS stack, due to its deformability, not simply a displacement shift. Thus, the influence of MA on a measured pulse signal is not simply a length change of the TCS stack but also a change in its system parameters (TVSPs), as detailed in Section 4.1.2. As such, compared to the current studies on pulse measurement and MA, the novelty of this work lies in a full consideration of the involvement of the TCS stack in pulse measurement.

#### 4.1.2. Existence of TVSPs

As shown in Figure 18, the presence of MA alters (1) the TCS stack length from $x_{DC}\;$ to $x_{DC}+\;x_{b}(t)$, and (2) the prestress in tissue, which results in TVSPs: m(t), k(t), and c(t). Note that $F_{b}(t)$ associated with $x_{b}(t)$ is equivalent to MA altering the contact pressure from $P_{c}$ to $P_{c}+F_{b}(t)$. In the structural dynamics field, it is well established that prestress in a solid alters its mechanical properties and consequently its system parameters when treated as a dynamic system [20]. Furthermore, a vibrator–ground system has been extensively studied for the influence of contact pressure on the response of the ground [15]. The TCS stack in pulse measurement is analogous to a vibrator–ground system [15]. While the system parameters of the ground are altered by the contact pressure, the system parameters of the tissue are altered by $x_{b}(t)$ or $F_{b}(t)$.

**Figure 19:**
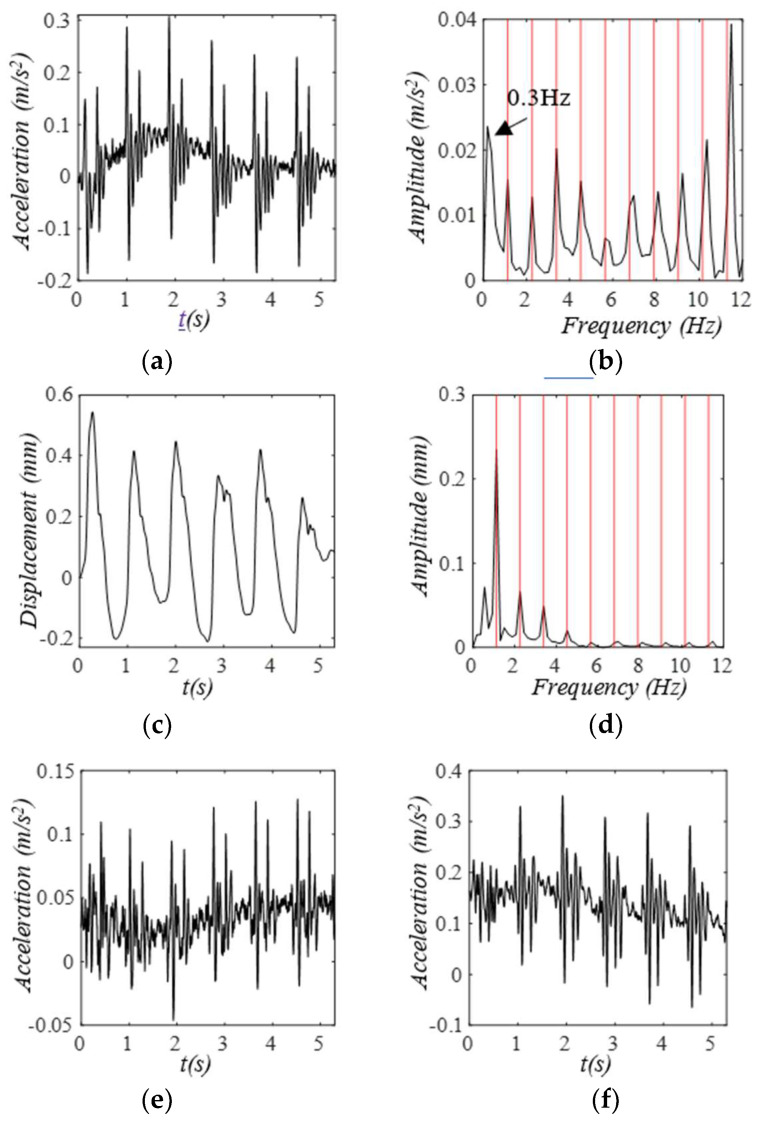
Measured pulse signals at the CA of a 30-year−old male subject using an analog accelerometer. (**a**) original recorded acceleration signal (x−axis). (**b**) its frequency spectrum. (**c**) derived displacement signal. (**d**) its frequency spectrum. (**e**) accompanying acceleration signals in the y−axis and (**f**) z−axis direction.

In addition to the above theoretical basis for the TVSPs, we further illustrate the existence of TVSPs with a measured pulse signal at the CA of a 30-year-old male healthy subject at rest using an analog accelerometer (under the IRB approval at Old Dominion University) at our lab, as shown in Figure 19. The original recorded acceleration signal captures all the pulse cycles. As evidenced by the large peak at 0.3 Hz, this acceleration signal suffers a high level of MA. Its frequency spectrum reveals all the peaks of its harmonics, but these peaks are smeared by adjacent low-amplitude signals. The displacement signal (no baseline drift removal) integrated from the acceleration (using the filtering technique for the acceleration and the velocity in [21]) shows varying APWs and varying HRs between pulse cycles, where TVSP-generated distortion is evidenced by low-amplitude signals centering around the first~fourth harmonics of the pulse signal in Figure 19d. As shown in Figure 19e,f, an accelerometer tilts along the other two directions in pulse measurement, leading to their own baseline drifts and conceivably contributing to the TVSPs of the TCS stack.

### 4.2. Baseline Drift Versus TVSP-Generated Distortion

While baseline drift, as additive noise, indicates the level of MA and is independent of the true pulse signal, the TVSPs are multiplied by the true pulse signal and thus present as multiplicative noise in a measured pulse signal. Despite being low frequency in nature, TVSPs ride on each harmonic of the true pulse signal and thus smear each harmonic’s information of the true pulse signal in a measured pulse signal. As such, TVSP-generated distortion, spanning from the first to the last harmonic of the true pulse signal, cannot be simply removed by filtering/EMD and CSE.

As shown in Section 3, the influence of TVSPs on a measured pulse signal varies with the true pulse signal used. When pulsatile pressure is used as the true one, TVSP-generated distortion is more severe, as opposed to arterial wall displacement as the true pulse signal. Although the influence of TVSPs on a measured pulse signal is not as obvious as that of baseline drift in the time domain, the influence of TVSPs on a measured pulse signal in the frequency domain becomes obvious, in the sense that each harmonic of the true pulse signal is smeared by the TVSPs, which is expected to affect the time-derivatives of a measured pulse signal to a much larger extent than the pulse signal itself [9]. Given the clinical value of the time-derivatives of a measured pulse signal [1,2,3,4], the influence of TVSPs on a measured pulse signal needs to be considered.

In addition to affecting the amplitude and APW of a measured pulse signal, baseline drift and TVSPs both affect the start/end of a pulse cycle and cause HR variations between pulse cycles. Thus, the HRV estimated from a measured pulse signal may contain the contribution from MA. Moreover, any variation in the start/end of a pulse cycle affects the estimation of baseline drift and the obtained APW [11].

Equivalent forces for MA analyzed here reveal the complexity that MA introduces into a measured pulse signal. In particular, when Δp(t) serves as the true pulse signal, two different equivalent forces need to act on the mass and the arterial wall simultaneously to provide the TVSP-generated distortion. Although these forces are complex in their frequency content, they allow the TCS stack to remain as a time-invariant 1DOF or 2DOF system, which might be useful for quantifying baseline drift and TVSP-generated distortion in the future. The accuracy of arterial indices derived from APW depends on its fine features, and any minor inaccuracy in APW may undermine the clinical value of its derived arterial indices. As shown above, baseline drift and TVSP-generated distortion affect these fine features, and thus, their accurate estimation is crucial for achieving the needed accuracy.

### 4.3. Comparisons with the Related Studies on PPG Signals

Due to their low cost and ease of use, PPG sensors have been widely used for pulse measurement [1,12,13,14,16]. New features and improvements in PPG sensors have been pursued for enhancing their pulse measurement [22,23]. In their early days, PPG sensors were mostly used at the index finger, and their measured pulse signals, commonly referred to as PPG signals, were extensively used to obtain amplitude, APW, and their time-derivatives for clinical values [1]. In recent years, PPG sensors have been used at other locations (e.g., wrist) aside from the index finger for monitoring HR and respiration rate (RR) at rest and during activities [12,13,16,24]. In fact, the majority of studies and data-processing algorithms on MA have focused on PPG signals [24]. It should be noted that the frequency (0.3~7 Hz) of MA during activities [16] falls into the frequency of the pulse signal itself, which is out of the scope of this study. Here, we relate this analytical model to the current studies on PPG signals and their MA at rest.

Optical transduction used in a PPG sensor greatly complicates the influence of MA on a measured pulse signal [2,16,21]. In addition to participating in the dynamic behavior in pulse measurement, the TCS stack is also involved in optical transduction. For simplicity, ambient light variation [2] and ambient light leakage at the skin surface [16,24] due to MA are not considered here. A reflection-mode PPG sensor is considered. A PPG sensor consists of a light source and a photodetector. The light source emits light that is absorbed by tissue and blood in an artery, and the photodetector measures the amount of reflected light at the skin surface. While tissue absorbs and scatters light, it is hemoglobin in blood that is the main light absorber. A PPG signal consists of a DC (non-pulsatile) component and an AC (pulsatile) component. The DC component results from tissue and blood at the start/end of a pulse cycle, and the AC component arises from the true pulse signal (blood volume change or radius change) in an artery. MA alters the optical path length (or TCS stack length) relative to the artery. The influence of this length change (baseline drift) on a PPG signal is included in the DC component, which is separated from the AC component representing the displacement at the arterial wall. Yet, due to the deformability of the TCS stack, the altered optical path length is not simply $x_{b}(t)$ but $x_{b}\left(t\right)+x_{TVSP}\left(t\right)$, as shown in Figure 18. It should be noted that the absorption property of the TCS stack is also altered by the change in optical path length. As shown in Equation (7b), to account for optical transduction, $g(x_{b}\left(t\right)+x_{TVSP}\left(t\right))$ serves as the influence of the total distortion on a measured pulse signal, where the function g(x) accounts for the complexity in optical transduction [16]. However, because of the frequency of $x_{TVSP}\left(t\right)$, $g(x_{TVSP}\left(t\right))$ appears in the AC component. Similarly, when pulsatile pressure serves as the true pulse signal, the DC component should be $x_{1b}\left(t\right)+x_{1TVSP}+g(x_{2b}\left(t\right)+x_{2TVSP}\left(t\right))$, and $x_{1TVSP}+g(x_{2TVSP}\left(t\right))$ appears in the AC component.

Since data-processing algorithms cannot accurately estimate MA in a PPG signal, reference sensors have been employed for capturing MA during pulse measurement [14,16]. An accelerometer usually fails to capture the low-level MA at rest with desired accuracy [16]. In addition to a PPG sensor for pulse measurement, a reference PPG sensor is added to capture MA [16]. Yet, the reference sensor and the PPG sensor sit on different TCS stacks, and thus, MA varies between the two sensors. This is analogous to alignment variation leading to different measured pulse signals.

Respiration in MA is different from other body motions and finger jittering. In the studies on extracting RR from a PPG signal, respiration has three effects on a PPG signal: amplitude modulation (AM), frequency modulation (FM), and baseline drift [12,16]. While AM and FM are related to cardiopulmonary physiology, respiration shown in baseline drift is due to its effect on body motion. It needs to be pointed out that the effects of AM and FM on a measured pulse signal are neglected when the APW and pulse amplitude are of interest [1,2,3,4,5,6,7,8,9,10,11]. Only when RR needs to be extracted from a measured pulse signal are these two effects considered [12,16].

AM, FM, and baseline drift are all explored for the extraction of RR [12]. It is found that FM-based extraction of RR is the most accurate, as compared to AM and baseline drift. This is simply due to the fact that the presence of other MAs affects the pulse amplitude and baseline drift to a greater extent than frequency in a measured pulse signal, especially when the max slope point (least sensitive to MA) in a pulse signal is used for the extraction of RR [12]. Moreover, in FM-based extraction, Chon et al. [13] developed VFCDM (varying frequency complex demodulation) to obtain the time-varying frequency of the fundamental harmonic in a pulse signal. Afterward, the variation in frequency was used to represent RR. It should be stressed that this time-varying frequency in VFCDM arises from the physiological effect of FM on an arterial pulse signal, irrelevant to MA. As shown in this study, respiration in MA, together with other motions in MA, leads to TVSPs and the distortion of each harmonic, which is not considered in VFCDM. Finally, it is worth noting that all the current data-processing algorithms on PPG signals have not considered TVSPs and their influence on the APW, HR, and RR.

### 4.4. Future Work for Quantification of MA and the TCS Stack

In the analytical model, there are six parameters: $m_{0}$, $k_{0}$, and $c_{0}$ (nominal values) and m(t), k(t), and c(t) in the TCS stack. While $m_{0}$, $k_{0}$, and $c_{0}$ are determined by the tissue, sensor, alignment, and *P_c_*, m(t), k(t), and c(t) are associated with $x_{b}(t)$. Currently, both the values of $m_{0}$, $k_{0}$, and $c_{0}$ and the relation of m(t), k(t), and c(t) to $x_{b}(t)$ are unknown. The values of $m_{0}$, $k_{0}$, and $c_{0}$ and the relation of TVSP $\sim x_{b}(t)$ have to be qualitatively assumed, based on the related experimental results [9]. As such, although the analytical model presented here offers the potential of quantifying the influence of MA on a measured pulse signal, further studies are needed to extract the six parameters of the TCS stack and baseline drift from a measured pulse signal.

When free of MA, three parameters, namely, $m_{0}$, $k_{0}$ and $c_{0}$ of the TCS stack, determine the harmonic-dependent transfer function from the true pulse signal to a measured pulse signal, as shown in Equations (8) and (19). However, the fundamental frequency and its harmonics in a measured pulse remain constant and the same as those in the true pulse signal. When MA appears, baseline drift and TVSPs are nonstationary (or non-harmonic) signals, and their manifestation in a measured pulse signal leads to a nonstationary signal. If forced to be treated as time-harmonic, a measured pulse signal has its instant (or time-varying) frequency and amplitude [18], as expressed in Equations (6) and (17). Thus, this analytical model falls into the system dynamics field, where a research topic under intensive study is time-frequency analysis of a measured signal from a dynamic system for identifying its time-varying system parameters [18]. To date, different approaches have been developed to extract the instant frequency and instant amplitude for obtaining time-varying system parameters from a single measured signal [18]. With this analytical model, such time-frequency analysis on a measured pulse signal might be implemented for ultimately quantifying the six parameters of the TCS stack and baseline drift.

### 4.5. The Fixing of an Accelerometer and a PPG Sensor

While PPG sensors are widely used for pulse measurement, only a few studies reported on accelerometers for pulse measurement at the CA [2,25,26]. The rare application of accelerometers for pulse measurement can be attributed to two reasons: (1) an accelerometer measures an acceleration signal, but the acceleration of a pulse signal lies at the bottom of the measurement range of the most-sensitive accelerometer [25,26]; and (2) the integral of the measured acceleration signal incurs great inaccuracy in the obtained displacement signal, due to random noise accumulated in the integral process [21].

As shown in Figure 1 and Figure 2, due to their great sensitivity, a PPG sensor and an accelerometer are usually fixed at an artery by tape; the fixing itself needs to move up and down, together with the sensor. As such, the fixing becomes part of the TCS stack. Then, other than presetting the nominal values of the TCS stack via exerting *P_c_*, the fixing forms part of the TCS stack and contributes to its system parameters. As shown in Equations (8) and (19), even when free of MA, the TCS stack is essentially a harmonic-dependent transfer function from the true pulse signal to the measured pulse signal. The fixing also contributes to this transfer function for the measured pulse signal and adds another hurdle to comparability between studies using different fixings.

### 4.6. y(t) Versus Δp(t) as the True Pulse Signal

Experimental studies reveal that the measured pulse amplitude increases when *P_c_* increases from low to high [2,9]. However, when *P_c_* is excessive, the measured pulse amplitude gets smaller, due to the suppression of the true pulse signal in an artery [2]. This suppression confirms the influence of *P_c_* on the true pulse signal in pulse measurement. It is reasonable to believe that both y(t) in Figure 1 and Δp(t) in Figure 2 are affected by the TCS stack during measurement, since the arterial wall and blood flow in it function as an inseparable entity. To the best of the authors’ knowledge, in all the literature on pulse measurement, the measured pulse signal with maximum amplitude is deemed as the most accurate measurement and is directly used to represent the true pulse signal in an artery for deriving arterial indices.

Here, y(t) as the true pulse signal represents the case where the true pulse signal in an artery is not affected during measurement. In contrast, to some extent, Δp(t) as the true pulse signal represents the case where the true pulse signal is affected during measurement, since y(t) is affected by the TCS stack, as shown in Equations (19a) and (19b). Yet, as explained above, Δp(t) and y(t) in an artery should vary simultaneously [17,19]. As such, the modeling of the arterial wall as a spring in Figure 2 is a rather simplified treatment for considering the influence of the TCS stack on the true pulse signal. The influence of MA on a measured pulse signal with Δp(t) as the true pulse signal is believed to better reflect reality. The calculated results in Section 3 reveal that the influence of the TCS stack on the true pulse signal leads to larger TVSP-generated distortion in a measured pulse signal, when the influence of *P_c_* on the true pulse signal is included. Meanwhile, this influence also causes higher deviations in a measured pulse signal free of MA from the true pulse signal, in terms of amplitude and APW.

As shown in Equation (19), the transfer function from the true pulse signal to the measured pulse signal is determined by the system parameters of the TCS stack, which are further determined by the tissue, the sensor used, as well as *P_c_* (or fixing). Experimental studies have noticed a great variation in the measured APW with *P_c_*, in addition to the measured pulse amplitude [2]. This variation associated with the contact pressure could play a role in (1) setting the system parameters of the TCS stack, as shown in this study, and (2) affecting the true pulse signal, as explained above. Overall, the influence of *P_c_* on a measured pulse signal can be factored into the TCS stack: the TCS stack affects the measured pulse signal via dictating the transfer function and affecting the true pulse signal.

Despite containing the effects of the transfer function and the affected true pulse signal, the measured pulse signals with maximum amplitudes are related to different CV conditions with statistical significance in numerous clinical studies [2,9]. This might imply that these two effects of the TCS stack generate similar deviations in measured pulse signals from true pulse signals between the two conditions, after they are averaged among individuals in each condition. However, in these studies, the standard deviations of arterial indices in each condition are usually even larger than the difference itself [2,3], which could possibly imply non-negligible variations in system parameters of the TCS stack between individuals. Because of the two effects of the TCS stack, it remains elusive to identify a fine difference in arterial indices between the two CV conditions and to achieve the reliability of arterial indices at the individual level. As examined here, the influence of MA adds another layer of complexity to the extraction of the true pulse signal from a measured pulse signal.

### 4.7. Study Limitations

There are five limitations in this study. Firstly, the TCS stack is assumed to behave linearly. This is a reasonable assumption in the sense that the measured pulse signals at the arterial wall by ultrasound share similarity in APW with those measured by sensors at the mass of the TCS stack. There may be some nonlinearity in the TCS stack due to its geometrical and anatomical complexity, but this nonlinearity may not be significant. Secondly, TVSPs in the TCS stack are assumed to vary linearly with baseline drift. Strictly speaking, the relation between them may not be linear, simply due to (1) the geometrical and anatomical complexity in the TCS stack and (2) MA experienced by a sensor in the other two directions (see Figure 19). Nevertheless, this assumed relation is qualitatively practical [9] in the sense that the TVSPs are expected to follow the varying trend of baseline drift, since baseline drift presets the stress in the TCS stack and then tunes its dynamic behavior [9,15,20]. Thus, the obtained influence of TVSPs on a measured pulse signal is qualitatively valid. Future studies need to be conducted to derive their quantitative relations from a measured pulse signal.

Thirdly, the true pulse signal is unaffected by the TCS stack. As explained above, the TCS stack might affect the true pulse signal in an artery, and the TCS stack further causes deviation of the measured pulse signal from the affected true pulse signal. It is this TCS stack that is the root cause of experimentally observed great variations between individuals and between measurements. Therefore, more work is needed to quantitatively evaluate the influence of the TCS stack on a measured pulse signal. Nevertheless, this study provides a deeper understanding of the influence of all the factors, especially MA, involved in pulse measurement on a measured pulse signal, which could serve as a fundamental framework for quantifying the influence of the TCS stack and MA on a measured pulse signal in the future. Fourthly, transduction (or noise) associated with a sensor itself is not considered. As mentioned earlier, optical transduction used in a PPG sensor is sensitive to ambient light variation and ambient light leakage at the skin surface [2]. A measurement by an accelerometer is subject to much higher noise due to the above-mentioned two reasons, as compared to a measurement by a tactile sensor, as will be seen in Part II. Lastly, no experimental studies have been conducted to quantitatively validate this model. Although the analytical model offers a quantitative relation between MA and a measured pulse signal, further time-frequency analysis is needed to extract the six unknowns of the TCS stack and baseline drift from a measured pulse signal, prior to quantitative validation.

## 5. Conclusions

In this paper, an analytical model of MA in a measured pulse signal by an accelerometer and a PPG sensor is presented. MA causes baseline drift and TVSPs simultaneously in the transmission path (i.e., TCS stack) from the true pulse signal to the measured pulse signal. Baseline drift represents a low-frequency signal and indicates the level of MA in a measured pulse signal. While TVSPs generate relatively abrupt distortion in a measured pulse signal, they can cause 1% variation in heart rate, 43% change in pulse amplitude, and, more importantly, they ride on each harmonic of the true pulse signal and distorts each harmonic in a measured pulse signal. As such, current data-processing algorithms based on the low-frequency nature of MA at rest are unsuitable for MA estimation.

As compared to the current studies on pulse measurement and MA, the novelty of this model lies in a full consideration of the involvement of the transmission path in pulse measurement, which identifies (1) the role of the TCS stack as a harmonic-dependent transfer function between a measured pulse signal and the true pulse signal and (2) the existence of TVSP-generated distortion in a measured pulse signal. Although this analytical model clearly defines the parameters that quantify the TCS stack and MA and provides a mathematical relation of MA to a measured pulse signal, further studies are needed on the time-frequency analysis of a measured pulse signal for obtaining the actual values of the parameters quantifying the TCS stack and MA. Finally, due to the motion of the accelerometer and the PPG sensor in pulse measurement, their fixing contributes to the nominal values of the transmission path and undermines comparability between studies where such fixing varies. In Part II of this work, an analytical model of MA in a measured pulse signal by a tactile sensor will be presented and compared with the findings here.

## Data Availability

The data that support the findings of this study are available upon reasonable request from the corresponding author. The data are not publicly available due to privacy or ethical restrictions.

## Abbreviations

The following abbreviations are used in this manuscript:

BD	Baseline drift
DOF	Degree-of-freedom
TVSP	Time-varying system parameter
MA	Motion artifact
TCS	Tissue–Contact–Sensor
HR	Heart Rate
